# Predictors of Nightly Subjective-Objective Sleep Discrepancy in Poor Sleepers over a Seven-Day Period

**DOI:** 10.3390/brainsci7030029

**Published:** 2017-03-09

**Authors:** Vanessa Herbert, Daniel Pratt, Richard Emsley, Simon D. Kyle

**Affiliations:** 1 Division of Psychology and Mental Health, School of Health Sciences, University of Manchester, Manchester Academic Health Science Centre, Manchester M13 9PL, UK; Vanessa.Herbert@postgrad.manchester.ac.uk (V.H.); Daniel.Pratt@manchester.ac.uk (D.P.); 2 Centre for New Treatments and Understanding in Mental Health (CeNTrUM), University of Manchester, Manchester Academic Health Science Centre, Manchester M13 9PL, UK; 3 Centre for Biostatistics, School of Health Sciences, University of Manchester, Manchester Academic Health Science Centre, Manchester M13 9PL, UK; Richard.Emsley@manchester.ac.uk; 4 Sleep and Circadian Neuroscience Institute (SNCI), Nuffield Department of Clinical Neuroscience, Sir William Dunn School of Pathology, University of Oxford, Oxford OX1 3PA, UK

**Keywords:** subjective/objective sleep discrepancy, misperception, insomnia

## Abstract

This study sought to examine predictors of subjective/objective sleep discrepancy in poor sleepers. Forty-two individuals with insomnia symptoms (mean age = 36.2 years, 81% female) were recruited to take part in a prospective study which combined seven days of actigraphy with daily assessment of sleep perceptions, self-reported arousal, sleep effort, and mood upon awakening. A high level of intra-individual variability in measures of sleep discrepancy was observed. Multilevel modelling revealed that higher levels of pre-sleep cognitive activity and lower mood upon awakening were significantly and independently predictive of the underestimation of total sleep time. Greater levels of sleep effort predicted overestimation of sleep onset latency. These results indicate that psychophysiological variables are related to subjective/objective sleep discrepancy and may be important therapeutic targets in the management of insomnia.

## 1. Introduction

Insomnia disorder is characterised by persistent difficulties with the initiation and/or maintenance of sleep, leading to daytime impairment [1,2]. Epidemiological studies have shown that approximately one in ten people meet the criteria for insomnia disorder [3,4]. Insomnia can have severe consequences for many aspects of life, including work performance, social functioning, and health [5,6,7,8,9,10].

A diagnosis of insomnia disorder is typically based on self-reported symptoms alone. However, numerous studies have demonstrated a mismatch between subjective reports and objective estimates of sleep in people with insomnia [11]. Specifically, many individuals with a diagnosis of insomnia disorder do not demonstrate sleep abnormalities according to objective assessments such as Polysomnography (PSG). Where objective measures do corroborate abnormalities in sleep, it is often not to the extent that the subjective report suggests [12,13,14,15,16]. There is debate in the field as to whether the subjective/objective mismatch represents a distinct subtype of insomnia (“sleep state misperception”, “subjective insomnia”, “paradoxical insomnia”), or is a more general feature of the disorder [11,14,15,17,18]. Whilst there is considerable variation in the accuracy of sleep perceptions within insomnia samples [14,15,19], on average, individuals with insomnia have a tendency to underestimate actual sleep [15,18]. This is in contrast to good sleepers, who tend to overestimate sleep [20,21,22,23] or estimate objective sleep parameters accurately [18,24,25]. These findings have implications for the assessment and diagnosis of insomnia and highlight uncertainties that exist around whether insomnia is best captured by subjective or objective methods. They also expose the potential importance of perceptions of sleep for our understanding of insomnia.

The discrepancy between subjective reports and objective estimates has been demonstrated in a variety of indices of sleep, including total sleep time (TST) [12,13,14,26], sleep onset latency (SOL) [13,24,27,28,29], wake-time after sleep onset (WASO) [24,28,29], and sleep efficiency (SE) [26,30]. Discrepancy occurs in primary insomnia and also in patient groups where insomnia is comorbid with other health or psychiatric conditions [31,32,33,34,35,36]. There is evidence that discrepancy increases with advancing age and may play a role in the higher rates of self-reported insomnia in later life [29].

The tendency for people with insomnia to underestimate objective sleep has been conceptualised in several ways; (i) as an exaggeration of sleep difficulties, perhaps due to more general psychological characteristics and personality traits [19,27]; (ii) as a meaningful phenomenon which reflects a localised sleep disturbance with candidate physiological markers [20,28]; and (iii) as a cognitive distortion which contributes to the maintenance and escalation of insomnia [37]. According to Harvey’s Cognitive Model (2002), those who underestimate their sleep may be more at risk of developing objective sleep deficits due to increased preoccupation with sleep and increased sleep related anxiety and arousal, which is antithetical to optimal sleep onset and maintenance [37]. In line with this model, studies which have sought to correct sleep discrepancy have demonstrated changes in insomnia related anxiety and distress and concomitant reductions in self-reported insomnia symptoms [38,39,40]. There is emerging evidence that Cognitive Behavioural Therapy for Insomnia (CBT-I) improves the accuracy of sleep perceptions [30], a finding which highlighted the possibility that correction of discrepancy could account for some of the efficacy of treatments for insomnia.

A variety of psychological and physiological mechanisms have been proposed to underlie sleep discrepancy (for a review see Harvey and Tang [11]). These include cognitive arousal [41,42], physiological arousal [41,42,43], cortical arousal [20,44], selective attention [45], memory bias [11], and sleep fragmentation [46,47]. A recent study by Takano and colleagues (2016) [48] in a community sample comprising individuals with and without insomnia symptoms, reported that higher levels of pre-sleep cognitive arousal were associated with underestimations of TST and overestimations of SOL. This work is consistent with correlational and experimental studies that have reported associations between pre-sleep cognitive activity and sleep discrepancy in insomnia samples [19,41].

The studies conducted thus far have assessed sleep discrepancy over a single night or averaged data from multiple nights. Therefore, little is known about intra-individual variability in sleep discrepancy and whether it is affected by natural, day-to-day variations in psychological factors. There is some evidence for high-levels of night-to-night variability in sleep discrepancy in older adults, where the discrepancy between self-report and actigraphy based estimates of sleep onset latency was found to be 150% more variable within the same individual across nights, compared to between individuals [29]. In addition, there is emerging evidence for intra-individual variability in some of those factors which are proposed to underlie discrepancy, such as arousal [49,50] and sleep fragmentation [51]. The importance of examining intra-individual variability in sleep/wake patterns is increasingly being recognized [52]. Although daily and average values of sleep parameters tend to be highly correlated, information may be concealed when only average or single values are considered. It is possible that daily values and averaged values have overlapping but distinctive aetiology. For example, daily values may be more highly associated with state like psychophysiological variables than average values.

In this study, we utilised multilevel modelling to investigate whether self-reported arousal (cognitive, physiological), sleep effort, sleep fragmentation, and mood upon awakening predicted sleep discrepancy across seven nights, in a group of poor sleepers reporting insomnia symptoms. We chose to investigate multiple constructs in order to tease out the most important contributors. Multilevel modelling allows for the analysis of within-person changes in variables across nights whilst accounting for the influence of between-subject variations in the relationships of interest. By using this approach, we sought to examine intra-individual variability in sleep discrepancy and assess the relationship between sleep discrepancy and psychophysiological variables over multiple nights, without the requirement to aggregate data [53]. We chose to focus on cognitive arousal and sleep fragmentation because these constructs have the strongest evidence as predictors of subjective/objective sleep discrepancy [11]. We included two measures of cognitive arousal; one that assesses general cognitive arousal and another that assesses the content and frequency of thoughts during the pre-sleep period, due to preliminary evidence that certain aspects of cognitive arousal may be more closely associated with sleep disturbance [54]. We included a measure of self-reported physiological arousal because an experimental study has shown that increases in physiological arousal lead to increases in TST and SOL discrepancy [41]. The inclusion of a measure of sleep effort was based on research demonstrating that sleep effort is strongly associated with subjective reports of sleep disturbance but not objective sleep parameters (PSG) [55]. Mood upon awakening was assessed due to evidence that low mood and general feeling state, at the time of reporting of subjective sleep, may mediate underestimations of objective sleep parameters [35,56]. It was hypothesised that higher levels of arousal (cognitive and physiological), sleep effort, sleep fragmentation, and worse mood on awakening would be associated with the overestimation of TST. It was also hypothesised that higher levels of arousal (cognitive and physiological), sleep effort, and worse mood on awakening would be associated with the overestimation of SOL. We focused on discrepancies in TST and SOL because the subjective/objective discrepancy has been demonstrated more robustly in these indices.

## 2. Materials and Methods

### 2.1. Procedure

Participants were recruited from the staff and student population of the University of Manchester and the local community, through advertisement. Ethical approval for the study was obtained from the University of Manchester Research Ethics Committee (UREC ID: 15224). Inclusion criteria were: (1) aged between 18 and 65 years and (2) score on the Sleep Condition Indicator (SCI) ≤16 indicating probable insomnia disorder. Exclusion criteria were: (1) sleep disorder other than insomnia; (2) current treatment for any medical or psychiatric disorder; (3) current use of hypnotic medication or medication that is known to cause drowsiness. People who were interested in taking part in the study were directed to a webpage which provided information about the study and screening questionnaires. The researcher contacted those participants who were deemed eligible according to their responses on the screening questionnaires. Each eligible participant attended the University for one face-to-face session with the lead researcher (VH), where they provided informed consent to take part in the study and completed a battery of baseline questionnaires. Participants were then provided with a Motionwatch 8 actigraph watch (CamNTech Ltd.; Cambridge, UK) in order to collect objective estimates of their sleep. Participants wore the watch continuously for a seven-day period. At the same time, participants recorded their subjective experiences of sleep by completing the Consensus Sleep Diary—Morning administration [57] each morning upon awakening. Alongside the sleep diary, participants completed retrospective assessments of their subjective experiences of general cognitive arousal, physiological arousal, sleep effort, and specific pre-sleep cognitive activity, for the preceding night. Finally, participants provided a rating for their current mood state. Participants received £5 as compensation for taking part in the study.

### 2.2. Screening Measures

All participants checked a box to endorse the inclusion and exclusion criteria. The Sleep Condition Indicator [58] was administered to assess insomnia symptoms. It was presented on the study webpage. It is an eight-item scale which evaluates SOL, WASO, sleep quality, impact of sleep problems on daytime functioning, duration of sleep problems, nights per week where a sleep problem is present, the extent to which the individual is troubled by the sleep problem, and the history of the sleep problem. Participants are asked to provide ratings with reference to a typical night in the past month. The SCI utilises a five-point scale (0–4), with lower scores indicating greater difficulties. Total score on the SCI ranges between 0 and 32, with scores ≤16 indicating probable insomnia [58]. The SCI has satisfactory internal consistency (Cronbach α = 0.86) [58] and correlates strongly with the Pittsburgh Sleep Quality Index (PSQI) [59] and the Insomnia Severity Index (ISI) [60]. A screening tool for the identification of sleep disorders other than insomnia, which was developed by experts in the field of sleep disorders [61], was also presented on the study webpage. Those scoring positive for potential narcolepsy, sleep breathing disorder, restless leg syndrome, circadian rhythm sleep disorder, or parasomnia were deemed ineligible to take part in this study.

### 2.3. Baseline Measures

Assessments of dysfunctional beliefs and attitudes about sleep, depression, anxiety, and stress were administered to characterise the sample. The Dysfunctional Beliefs and Attitudes about Sleep Scale (DBAS-16) [62] was administered to evaluate participants’ beliefs about sleep, specifically their views on the causes, consequences, and treatment of sleep problems. The scale is a brief form of the original 30 item DBAS and contains 16 items which are rated on a 11-point Likert scale ranging from 0 (strongly disagree) to 10 (strongly agree). Total scores are averaged to create a mean item score, with higher mean scores indicating stronger endorsement of maladaptive beliefs about sleep. The DBAS-16 has adequate internal consistency (Cronbach α = 0.77) [62] and good test-retest reliability (*r* = 0.83) [62]. In this sample, internal consistency was good (Cronbach α = 0.84).

Participants also completed the Depression, Anxiety and Stress Scale (DASS-21) [63] in order to provide a measure of symptoms of depression, anxiety, and stress over the past week. Each item is scored from 0 (did not apply to me at all over the last week) to 3 (applied to be very much or most of the time over the last week). This scale has been validated for non-clinical samples, where it demonstrated adequate internal consistency (Cronbach’s α = 0.88 for depression, 0.82 for anxiety, 0.90 for stress, and 0.93 for the total scale) and good convergent and discriminant validity when compared with other measures of anxiety and depression [64]. In this sample, internal consistency was satisfactory (Cronbach α = 0.93 for depression, 0.83 for anxiety, 0.87 for stress, and 0.94 for the total scale).

### 2.4. Daily Measures

#### 2.4.1. Actigraphy

Participants wore a MotionWatch 8 actigraphy watch, which is a tri-axial, wrist-worn accelerometer. The digital accelerometer enables the differentiation of probable sleep and wake states for each 30 s period of recording using a sleep/wake algorithm. Actigraphy has been validated against PSG, where it has shown high levels of sensitivity for detecting sleep [65]. Participants were asked to press the event marker when they got into bed at night and again when they got out of bed the following morning. Data were downloaded and analysed using Motionware 1.1.20 software (CamNtech Ltd., Cambridge, UK). Responses from the sleep diary were used to set the sleep period analysis window (i.e., between the “got into bed” and “got out of bed” times). In cases where these data were missing from the sleep diary, we used the event markers to mark the analysis window. This occurred for less than 1% of data points. The following sleep variables were extracted: TST, SOL, SE, and sleep fragmentation index. TST is the total time spent in sleep according to the epoch-by-epoch wake/sleep categorisation. SOL is the time which elapsed between the participant getting into bed and the participant falling asleep. SE is the total sleep time, expressed as a percentage of the total time spent in bed. The sleep fragmentation index is a percentage of the total time categorised as mobile in the epoch-by-epoch mobile/immobile categorisation and the number of immobile bouts which were less than or equal to one minute in length. The sleep fragmentation index is a measure of the degree of sleep discontinuity.

#### 2.4.2. Sleep Diary

This diary of subjective sleep experiences is widely used in sleep research and was developed through collaboration between a panel of experts in the field of insomnia and potential users of the diary [57]. Items directly assess or permit the calculation of total time in bed, total sleep time, sleep onset latency, wake-time after sleep onset, and sleep quality. Subjective TST was taken from participants’ estimates of total sleep time. Subjective SOL was calculated from the time that the participant got into bed using the following formula: (Time at which the individual attempted to fall asleep + self-reported latency to sleep)—Time at which the individual got into bed.

#### 2.4.3. Predictors of Discrepancy

Each morning participants completed four visual analogue scales (0–100 mm, with anchors “Not at all” and “Very Much”), assessing general cognitive arousal (“Last night, as you were attempting to fall asleep or return to sleep, did thoughts keep running through your mind?”) and physiological arousal (“Last night, as you were attempting to fall asleep or return to sleep, did you experience a jittery nervous feeling in your body?”). These questions were adapted from items on the Pre-Sleep Arousal Scale [66] and the same or similar single item questions have been employed in previous daily diary designs [67,68]. Sleep effort was assessed using an item adapted from the Glasgow Sleep Effort Scale [69], (“How much effort did you put into sleeping last night?”, with anchors “No effort” at 0 mm and “A lot of effort” at 100 mm). Current mood state was assessed by asking participants, “How would you describe your mood right now?” with anchors “very bad mood” (0 mm) and “very good mood” (100 mm). Participants completed the Glasgow Content of Thoughts Inventory (GCTI) to provide a retrospective measure of the content and frequency of pre-sleep cognitive activity. The GCTI was completed in the morning with reference to cognitive activity during the preceding night. The Inventory consists of 25 items which were developed on the basis of live audio recordings of pre-sleep thought content in people with insomnia. Participants were requested to indicate on a 4-point scale (1 = Not at all, 2 = A little, 3 = A fair amount, 4 = A great deal) to what extent specific thoughts kept them awake the previous night. Scores were summed and higher scores indicate a greater level of pre-sleep cognitive activity. The inventory has demonstrated good test-retest reliability (Intraclass correlation coefficient = 0.88) and satisfactory internal consistency (Cronbach α = 0.87). In a validation study, scores on this scale successfully discriminated between individuals with insomnia and good sleepers, with a score of 42 yielding a sensitivity of 100% and a specificity of 83% [70]. In the current sample, internal consistency for the total score was good (Cronbach α = 0.91 for day 1, 0.91 for day 2, 0.89 for day 3, 0.90 for day 4, 0.92 for day 5, 0.91 for day 6, and 0.92 for day 7).

### 2.5. Data Analysis

A TST discrepancy score was calculated using the formula outlined by Manconi and colleagues (known as the misperception index (MI) [18]; (objective TST—subjective TST)/objective TST. MI values range between +1 and −1. Perfect correspondence between subjective and objective estimates of TST results in an MI equal to 0. Positive MI values indicate underestimation of objective sleep and negative MI values indicate overestimation of objective sleep. The discrepancy score for SOL (SOL_d_) was calculated using the following formula: SOL_d_ = objective SOL—subjective SOL. SOL_d_ is positive when SOL is underestimated compared to objective values, and negative when SOL is overestimated compared to objective values. Descriptive statistics were computed to examine the distribution of the outcome variables (Table 1).

All analyses were performed in Stata, version 14 (Statacorp, 2015, College Station, TX, USA). All outcome and predictor variables were assessed for normality via histogram inspection and analysis of skewness and kurtosis statistics. SOL_d_ was negatively skewed and scores for the GCTI, general cognitive arousal, physiological arousal, sleep effort, and sleep fragmentation were positively skewed. Therefore, subsequent analyses used nonparametric bootstrapping with 200 replications to account for non-normality of the data. Due to the hierarchical nature of the data (observations are nested within participants), the assumption of independence of observations is violated and therefore multilevel modelling was used. Linear mixed effects models (“xtmixed”) were estimated using maximum likelihood estimation. For each analysis, the participant number was included as a random intercept. Two sources of variance were partitioned in the dataset; differences between participants on average levels of daily variables and difference within participants on their daily reporting of variables over time. In order to examine intra-individual variability in the sleep discrepancy variables over time, we estimated the intraclass correlation coefficient using the xtmixed post-estimation function (“estat icc”). The intraclass correlation quantifies the relative magnitude of the within and between person variance components in a multilevel model and was calculated for all sleep variables separately. To examine associations between sleep discrepancy variables and psychophysiologic predictor variables, univariate models were estimated for each predictor variable. Significant univariate predictors were then entered into a multivariable model in order to assess their contribution. Separate models were estimated for MI and SOL_d_.

## 3. Results

### 3.1. Participants

One-hundred and fifty-one self-reported poor sleepers completed the screening questionnaires. Of these, 45 were excluded because their score on the SCI did not indicate probable insomnia disorder. Thirty-eight people were excluded due to a possible sleep disorder (other than insomnia), three due to a psychiatric disorder, one person was excluded due to use of hypnotic medication, and one person was excluded due to use of a medication which may cause drowsiness. Of the 63 participants who were eligible to take part, 42 were enrolled in the actigraphy phase of the study. The most commonly cited reason for eligible participants not taking part was scheduling difficulties. Objective sleep data were not available for one participant due to malfunctioning of the actigraph watch. Therefore the final sample comprises data from 41 participants. Of a possible 287 assessment points for subjective and objective sleep, a total of 280 (98%) were completed.

Demographic and baseline characteristics of the sample are displayed in Table 2. Mean score on the SCI was 10.95, which is within the probable insomnia range. Eighty-three percent of participants reported enduring problems with sleep (>1 year). Mean score on the DBAS is similar to that seen in insomnia samples [62]. Although we excluded participants with a diagnosed psychiatric disorder, mean scores on the three subscales of the DASS-21 indicate high levels of depressive, anxiety, and stress symptoms in this sample compared to normative data from the general population (depression = 91st percentile, anxiety = 92nd percentile, stress = 96th percentile). The proportion of participants whose scores were in the severe or extremely severe range differed according to the subscale (depression subscale = 26.8%, anxiety subscale = 26.3%, and stress subscale = 55%). Mean estimates of sleep parameters are shown in Table 1. Actigraphy and sleep diary values for SOL and SE are similar to those reported for insomnia disorder samples in other published work. Estimates of TST appear to be slightly higher in this sample [71,72,73].

### 3.2. Misperception Index

Descriptive statistics for MI are presented in Table 1. The mean value for MI was 0.07, indicating that participants estimated on average 7% of objective sleep as wake. There was considerable intra-individual variability in MI, with differences within individuals accounting for 54.1% of the variance in MI. Variance and intraclass correlation coefficients for MI are presented in Table 3.

### 3.3. Daily Predictors of Misperception Index

Univariate analyses showed that the total score on the GCTI was a significant predictor of MI, with higher levels of pre-sleep cognitive activity predicting more positive values for MI (underestimation of total sleep time). General cognitive arousal, sleep effort, and sleep fragmentation index were also significant univariate predictors of MI, with higher values on these variables predicting more positive values for MI (underestimation of total sleep time). Self-reported physiological arousal was not a significant predictor. Mood on awakening was a significant predictor of MI, with better mood on awakening predicting more negative values for MI (overestimation of total sleep time). The coefficients, standard errors, confidence intervals, and significance values for each of the predictors in the univariate analysis are displayed in Table 4.

A multivariable analysis of the univariate predictors revealed that scores on the GCTI and mood upon awakening were significant independent predictors of MI. Specifically, worse mood upon awakening and higher levels of pre-sleep cognitive activity predicted more positive values for MI (underestimation of sleep time) (Table 5).

### 3.4. Sleep Onset Latency Discrepancy

Descriptive statistics for SOL_d_ are presented in Table 2. Differences within individuals accounted for 82.9% of the variance in SOL_d_. Variance and intraclass correlation coefficients for SOL_d_ are presented in Table 3.

### 3.5. Daily Predictors of Sleep Onset Latency Discrepancy

The univariate analyses showed that sleep effort was the only statistically significant predictor of SOL_d_. Higher values of sleep effort were associated with more negative values for SOL_d_ (Table 6), indicating that greater sleep effort predicts overestimation of the time taken to fall asleep. Since only one univariate predictor of SOL_d_ was identified, a multivariable analysis was not conducted.

## 4. Discussion

A mismatch between subjective and objective estimates of sleep parameters is commonly observed in people with insomnia, however little is known about the mechanisms underlying this phenomenon. This study sought to determine predictors of subjective/objective sleep discrepancy in individuals with insomnia symptoms. Using actigraphy and sleep diaries, we conducted repeated longitudinal assessments of sleep discrepancy, pre-sleep, and next-day psychophysiological factors, across seven days and nights. Our results highlight roles for arousal, sleep effort, mood upon awakening, and sleep fragmentation.

Examination of subjective and objective sleep over multiple nights enabled us to identify high levels of intra-individual variability for MI and SOL_d_ in this sample. Overall, 54.1% of the variation in MI was due to differences between days within the same participant. Both overestimation and underestimation of TST was evident. Of the 41 poor-sleepers who took part in the study, 30 (73%) displayed a mixture of over and underestimation of TST. These results do not support the proposal that underestimation of TST is a consistent and trait-like feature of people with insomnia. There was also considerable intra-individual variability in SOL_d_, where 82.9% of the variation was due to differences between days within the same participant. Both overestimation and underestimation of SOL was evident, however the frequency of underestimation of SOL was relatively small, with just 14.7% of subjective SOL values representing an underestimation.

### 4.1. Predictors of Subjective/Objective Sleep Discrepancy

Univariate analyses revealed that cognitive arousal (general cognitive arousal and specific pre-sleep cognitive activity measured using the GCTI), sleep effort, sleep fragmentation, and mood upon awakening were all significant predictors of MI. Multivariable analysis revealed that pre-sleep cognitive activity and mood upon awakening provided statistically significant, independent contributions to MI. With regards to SOL_d_, the univariate analyses identified sleep effort as the only significant predictor.

These findings suggest that cognitive arousal is associated with subjective/objective discrepancy in TST. These data corroborate evidence from an experimental study in which provoking an increase in cognitive arousal led to increases in TST sleep discrepancy [41]. They also support the work of Takano and colleagues [48], who found that cognitive arousal was uniquely associated with TST discrepancy in a community sample. Mechanisms through which cognitive arousal contributes to sleep discrepancy have been proposed, however the evidence is limited. One suggestion is that cognitive arousal distorts the perception of time because a unit of time is perceived as longer when more information is processed (through greater levels of mentation under high arousal conditions) [41]. Another proposal is that cognitive arousal maintains an enhanced level of sensory and memory processing during sleep onset, which obscures the distinction between sleep and wakefulness [20]. In line with this, an association between high-frequency Electroencephalogram (EEG) activity during non-rapid eye movement (NREM) sleep and subjective/objective sleep discrepancy has been observed [20,74]. High frequency EEG activity is thought to be a marker of sensory processing and memory formation. Further research implementing the fine grained measurement of sleep using techniques such as high density EEG is required to advance our understanding of the possible neurophysiological processes underlying associations between cognitive arousal and sleep discrepancy.

In this study, cognitive arousal was assessed using two measures; the GCTI which evaluates the content and frequency of pre-sleep cognitions and a visual analogue scale rating the extent to which participants experienced thoughts running through their minds (general cognitive arousal) during the pre-sleep period. In the univariate analyses, both measures of cognitive arousal were significant predictors of MI, however in the multivariable analysis, only the GCTI was a significant predictor. Clearly there is substantial overlap between these two measures, as indicated by the moderate strength correlation between responses (*r* (266) = 0.63, *p* < 0.001). Shared variance may explain why only the GCTI was a significant predictor of MI in the multivariable analysis, however there is little change in the predictive value of general cognitive arousal when scores on the GCTI were omitted from the multivariable analysis. The GCTI probes a wide variety of intrusive thoughts which are known to be commonly experienced by individuals with insomnia in the pre-sleep period and contains items such as “How frustrated/upset I am feeling” and “How nervous/anxious I am feeling”, which may capture the emotional and physiological sequelae of intrusive thoughts in a way that a single question about a racing mind does not. Although the GCTI is predominantly a measure of cognitive arousal, it appears to tap into hyperarousal more broadly and this may be the reason that it is the strongest predictor of MI in this study.

The findings from this study suggest that self-reported physiological arousal is not related to subjective/objective discrepancy in TST or SOL. This is contrary to reports from a previous study in which manipulations of physiological arousal using caffeine have led to changes in sleep discrepancy [41] and a study in which physiological arousal was shown to predict discrepancy in SOL [48]. We implemented a single item, self-report assessment of physiological arousal, which demonstrated sufficient sensitivity to detect associations between physiological arousal and various subjective and objective sleep parameters in a previous study of chronic pain patients [68]. However, self-report may be less sensitive in the domain of physiological arousal. Future work should administer validated subjective and objective measures of physiological arousal to fully assess its contribution to sleep discrepancy.

Mood upon awakening was a significant predictor of MI in both the univariate and multivariable analyses. Previous work has revealed associations between sleep discrepancy and depressive symptoms assessed at baseline [75,76]. The findings from this study extend that work by showing that daily fluctuations in morning mood are associated with subjective/objective discrepancy in TST. The relationship between sleep discrepancy and mood upon awakening may be explained by mood congruent memory bias, in which an individual recalls or selectively processes information that is consistent with their current mood. This is a phenomenon which has been documented in individuals with clinical depression [77] and during depressed mood induction in non-clinical populations [78]. Specifically, when an individual is making a judgement about how well they slept the previous night, current feeling state may distort memory such that low mood or dysphoria at the time of reporting leads to negatively biased judgements of sleep quantity and/or quality. It has long been established that memory is a reconstructive process affected by bias and error [79,80]. Moreover, mood congruent memory biases have been demonstrated in a variety of contexts, including symptom reporting [81,82].

Another possible explanation for the association between sleep discrepancy and mood upon awakening is that greater sleep discrepancy leads to worse mood upon awakening or that these variables are related by means of a third factor, such as sleep quality. An association between sleep quality and next-day affect is well established [83,84] and a number of studies have suggested a link between poor sleep quality and increased subjective/objective sleep discrepancy [28,30]. Due to the nature of the study design, causal inferences with regards to the relationships uncovered cannot be made. Experimental investigations are required to determine the direction of the effect and potential mediators of the relationship. For example, future studies could use a mood induction paradigm to examine the impact of mood upon awakening on subsequent subjective reports of sleep quantity and quality.

Consistent with our hypothesis, sleep effort was a significant predictor of both MI and SOL_d_. These findings lend support to the attention-intention-effort model of insomnia [85] which proposes that explicit intention to sleep inhibits normal de-arousal and subsequently hinders sleep. Sleep effort appears to play a particularly important role in SOL discrepancy, where it was the only significant predictor. In line with our findings, a previous study reported that reductions in sleep effort mediated the improved accuracy of sleep perceptions following paradoxical intention [86]. One possibility is that sleep effort maintains and exacerbates sleep difficulties through distorting perceptions of sleep.

Finally, this study revealed a significant association between sleep fragmentation (assessed by actigraphy) and MI in the univariate analysis, whereby higher levels of sleep fragmentation were associated with underestimation of TST. These findings concur with reports from an experimental study, in which inducing brief awakenings led to overestimates of sleep onset latency in normal sleepers [46]. More frequent awakenings may lead to shallower forms of sleep and greater levels of cortical activity, resulting in difficulties distinguishing wake from sleep [87].

### 4.2. Limitations

This study has several limitations. First, we examined associations between variables and therefore no causal inferences can be made. Second, potentially overlapping constructs were assessed, as indicated by moderate/strong correlations between many of the predictor variables. This complicates the interpretation of the results from the multivariable analysis. Third, the use of actigraphy enabled the assessment of sleep across multiple days in the home environment which increases the ecological validity of findings, however actigraphy is known to overestimate sleep time in individuals with insomnia [88]. This has implications for the reliability of the sleep discrepancy outcome variables. Fourth, our sample consisted predominantly of females, which limits the generalisability of the results. Fifth, we did not assess sleep microstructure and therefore the contribution of EEG parameters that are proposed to play a role in sleep discrepancy were not examined. Sleep discrepancy has been associated with heightened brain activity during PSG defined sleep [44]. Brief arousals from Rapid Eye Movement (REM) sleep and time spent in REM sleep have also been shown to correlate with the degree of discrepancy [89]. It will be important for future studies to include measures such as high density EEG, to understand how the relationships uncovered in the current study are expressed across different levels of explanation (i.e., underlying physiological processes). Finally, we did not conduct rigorous screening for sleep, physical health, or psychiatric comorbidities and we did not assess whether participants were taking substances that might induce sleeplessness (e.g., medications, caffeine, alcohol, illicit drugs). Therefore, it is possible that these factors influenced our findings.

## 5. Conclusions

This is the first study to examine intra-individual variability in sleep discrepancy and explore associations between sleep discrepancy and various psychophysiological factors using a repeated, longitudinal assessment in individuals with insomnia symptoms. High levels of intra-individual variability in estimates of sleep discrepancy were demonstrated, which supports the assertion that single measurements or aggregated measures of sleep discrepancy are likely to provide an incomplete picture. Associations between arousal, sleep effort, mood upon awakening, sleep fragmentation, and sleep discrepancy were identified. In a multivariable analysis, cognitive arousal and mood upon awakening were independently predictive of the total sleep time misperception index. Discrepancy in sleep onset latency was predicted by sleep effort. Further research is required to model possible additive and interactive effects among predictor variables.

## Figures and Tables

**Table 1:** Diary and actigraphy sleep parameters across study nights.

	Data Type	*n*	Mean	Min	Max	SD
TST	Subjective	281	391.27	135	880	89.34
	Actigraphy	281	425.13	142	759	73.12
SOL	Subjective	280	59.49	0	480	57.96
	Actigraphy	281	27.69	0	311	36.44
SE	Subjective	278	74.92	30.34	100	15.15
	Actigraphy	281	81.13	51.20	96.50	7.36
MI	-	280	0.07	−0.48	0.64	0.18
SOL_d_	-	279	−31.86	−445	73	51.18

SD = standard deviation, TST = total sleep time, SOL = sleep onset latency, SE = sleep efficiency, MI = misperception index, SOL_d_ = sleep onset latency discrepancy. TST and SOL were measured in minutes. SE represents time asleep as a % of total time in bed.

**Table 2:** Demographic and clinical characteristics.

Variable	Mean	SD
Age (years)	36.15	14.01
Sex (% female)	81	-
SCI	10.95	3.07
DBAS	4.86	1.29
DASS—Depression	7.80	7.15
DASS—Anxiety	5.98	5.22
DASS—Stress	13.73	7.05

SD = standard deviation, SCI = Sleep Condition Indicator, DBAS = The Dysfunctional Beliefs and Attitudes about Sleep Scale, DASS = Depression, Anxiety and Stress Scale

**Table 3:** Variance and intraclass correlation coefficients for sleep variables.

	Data Type	Between Person Variance (Intercept)	Within Person Variance (Residual)	Total Variance	ICC
TST	Subjective	2289	5663	7952	0.2879
	Actigraphy	1118	4215	5333	0.2096
SOL	Subjective	881.2	2474	3355	0.2627
	Actigraphy	308.7	1012	1321	0.2337
MI	-	0.015	0.018	0.033	0.4592
SOL_d_	-	445.6	2166	2611	0.1707

TST = total sleep time, SOL = sleep onset latency, SE = sleep efficiency, MI = misperception index, SOL_d_ = sleep onset latency discrepancy, ICC = intraclass correlation coefficient. TST and SOL were measured in minutes.

**Table 4:** Results from the univariate mixed model analysis predicting MI.

Predictors	C	95% CI	SE	*P*	*n*
GCTI	0.0059	0.0039, 0.0080	0.0010	<0.001	267
Cognitive Arousal	0.0012	0.0004, 0.0020	0.0004	0.003	277
Physiological Arousal	0.0004	−0.0005, 0.0012	0.0004	0.38	278
Sleep Effort	0.0017	0.0009, 0.0026	0.0004	<0.001	278
Sleep Fragmentation	0.0027	0.0010, 0.0044	0.0009	0.002	280
Mood	−0.0023	−0.0033, −0.0013	0.0005	<0.001	278

C = coefficient of the predictor, 95% CI = 95% confidence intervals, SE = standard error, *P* = significance level, *n* = number of observations, GCTI = Glasgow content of thoughts inventory, Mood = mood upon awakening.

**Table 5:** Results from the multivariable mixed model analysis predicting MI.

Predictors	C	95% CI	SE	*P*	*n*
GCTI	0.0038	0.0010, 0.0066	0.0014	0.007	265
Cognitive Arousal	−0.0004	−0.0015, 0.0006	0.0005	0.448	265
Sleep Effort	0.0008	−0.0003, 0.0020	0.0006	0.160	265
Sleep Fragmentation	0.0016	0.0002, 0.0035	0.0009	0.076	265
Mood	−0.0014	−0.0026, −0.0003	0.0006	0.024	265

C = coefficient of the predictor, 95% CI = 95% confidence intervals, SE = standard error, *P* = significance level, *n* = number of observations, GCTI = Glasgow Content of Thoughts Inventory, Mood = mood on awakening.

**Table 6:** Results from the univariate mixed model analysis predicting SOL_d_.

Predictors	C	95% CI	SE	*P*	*n*
GCTI	−0.5549	−1.2741, 0.1642	0.3669	0.130	266
Cognitive Arousal	0.0088	−0.2596, 0.2772	0.1369	0.949	276
Physiological Arousal	0.0427	−0.2288, 0.3141	0.1385	0.758	277
Sleep Effort	−0.2974	−0.5470, −0.0477	0.1274	0.020	277
Mood	0.3877	−0.0239, 0.7992	0.2100	0.065	277

C = coefficient of the predictor, 95% CI = 95% confidence intervals, SE = standard error, *P* = significance level, *n* = number of observations, GCTI = Glasgow Content of Thoughts Inventory, Mood = mood upon awakening.
